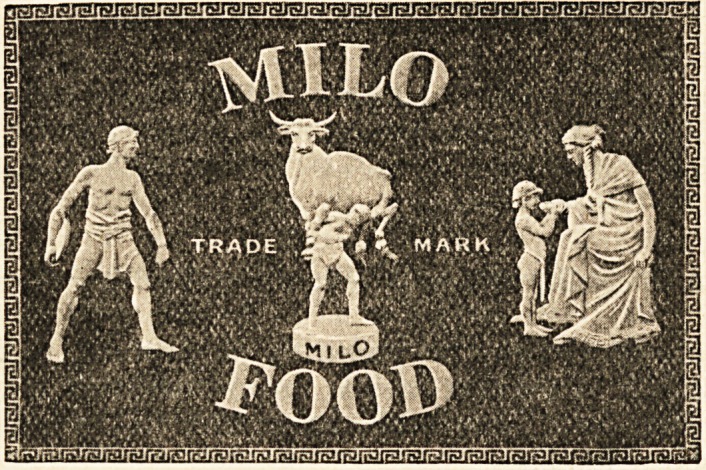# Notes on Preparations for the Sick

**Published:** 1904-12

**Authors:** 


### Notes on Preparations for the Sick.

For the various analyses contained herein we are indebted to Mr. Oliver C. M. Davis, B. Sc., A.I.C., of the University College, Bristol.

**Awards at the St. Louis Exhibition.**—Gratifying evidence of the recognition extended to British drug firms is furnished by the honours awarded by the Committee of the St. Louis Exhibition to Messrs. Burroughs, Wellcome & Co.'s exhibit of "Wellcome" Brand Chemicals, "Tabloid" and other pharmaceutical products and "Tabloid" Medical Equipments. Three grand prizes and three gold medals have been conferred for the scientific excellence of these products, and in recognition of the importance and educational value of the chemical and bacteriological researches conducted in the laboratories of this firm.

---

**Galak (Dry Milk).**—Galak Milk Products Limited, 118 Fenchurch Street, London, E.C.

This new dry sterile milk powder is one of the best of the various milk preparations at present on the market. A chemical examination shows that it is what it purports to be. It contains a good proportion of proteid, fat, and milk sugar, and forms an opalescent emulsion with water. It is pleasant to taste, and should prove a valuable addition to the dietary of invalids and others.

It is in the form of a light yellowish, flaky powder, which has the natural taste and odour of milk. It is obtained by drying milk rapidly at a high temperature, and is easily brought back to liquid milk of excellent quality by adding water. Milk to be dried by this process is fed continuously between two steam-heated cylinders revolving inversely and having a surface temperature in excess of 212° F. It passes between the cylinders (which are slightly separated), and is spread out in a thin uniform layer or film upon each cylinder and exposed thereon until reduced almost to dryness, being removed by a knife-edge while the film of milk solids yet retains sufficient moisture for their preservation. The milk solids come off the drying rolls in continuous moist sheets, which become dry instantly upon cooling, and are easily reduced to a uniform powder by being passed through a sieve.

The whole operation of evaporating the water of milk and obtaining its solids in dry conservable form occupies less than thirty seconds. The milk is dried as soon as it comes from the cow, and there is, therefore, no possibility of chemical change taking place in it before drying.

**Milo Food, for Infants and Invalids.**—Henri Nestlé, Vevey, Switzerland, and 48 Cannon Street, London.

The analysis of this food is given as follows:—

| | |
|---|---|
| Fat ... ... ... ... ... | 5.26 |
| Proteids ... ... ... ... ... | 14.02 |
| Sugar of Milk ... ... ... ... | 6.38 |
| Maltose and Dextrine ... ... ... | 26.12 |
| Cane Sugar ... ... ... ... | 27.67 |
| Starch ... ... ... ... ... | 14.95 |
| Mineral Matter ... ... ... ... | 1.95 |
| Water ... ... ... ... ... | 3.65 |
| | 100.00 |

It is supplied in hermetically-sealed tins, and is stated to be prepared from wheat, milk, and sugar only.

An examination shows it to be a cooked farinaceous food, containing in addition about 12 per cent. proteid and 5 per cent. fat. It should therefore be a nutritious food in cases where a rather large amount of carbohydrates is not objectionable.

The amount of starch present would, however, render it undesirable as an adjunct to the food of very young infants.

---

**Moseleys Food.**—Moseleys Food Limited, Stockport.

Is described as "a delightful supper dish," and "made in a minute," for infants, invalids and adults. A statement is made that it contains no "raw" starch, and no "insoluble" starch.

An examination shows that the preparation, although cooked, contains numerous grains which give the characteristic microchemical reactions of starch. It contains over 10 per cent. proteids, and a small amount of fat. The published analysis states that it contains about 60 per cent. dextrines and other soluble starch products, but our analysis differs with regard to this, as starch-grains cannot be considered as soluble products.

---

**Mellin's Food Biscuits.**—MELLIN'S FOOD LIMITED, Peckham London, S.E.

These biscuits contain a good proportion of the well-known Mellin's Food. They contain proteid matter, and also starch, and, being very portable, should be useful where a concentrated nutriment is desired.

---

**Mellin's Lacto-Glycose.**—(Mellin's Food with exsiccated fresh milk.)

An analysis shows the presence of proteid, dextrine, and fat. No starch is present, hence the preparation may be given to young infants. The following is the full analysis of the Lacto-Glycose food:—

| | |
|---|---|
| Moisture at 100° C. ... ... ... ... | 3.808 |
| Ash (containing 0·827 $P_2O_5$) ... ... | 3.640 |
| Fat ... ... ... ... ... ... | 4.579 |
| Carbohydrates— | |
| (*a*) Maltose and Lactose ... ... | 56.319 |
| (*b*) Dextrine ... ... ... ... | 18.162 |
| (*c*) Cellulose, Fibre, &c. ... ... | 0.800 |
| Proteids— | |
| (*a*) Albumin (containing 1.51 N.) ... | 9.437 |
| (*b*) Peptones (containing 0.18 N.) ... | 1.120 |
| (*c*) Insoluble and Amide Nitrogenous matter (containing 0.302 N.) ... | 1.887 |
| Unestimated... ... ... ... ... | 0.248 |
| | 100.000 |

For use it should be mixed with warm water only; thus prepared it constitutes an easily-digested, nutritious, and sustaining food for the old as well as the young. This combination of carbohydrate with proteid is really an improvement on an ordinary milk food; it is therefore something more than a substitute for milk when the latter cannot be obtained or cannot be guaranteed as free from injurious qualities. Its nutritive value is superior to that of milk alone, and, being soluble, it is not likely to cause any digestive troubles.

---

**Cheltine Foods: Diabetic Food; Almond Meal Biscuits; Soluble Milk Food.**—CHELTINE FOODS LIMITED, Cheltenham.

We have directed attention to some of the Cheltine foods on a previous occasion (vol. xxi., p. 373). A pamphlet issued by the Cheltine Company dilates on the value of the CHELTINE DIABETIC FOOD for diabetics, and states that the contained starch, though it gives the usual reactions, exists in a modified form. The preparation is undoubtedly cooked, but micro-chemical examination shows the presence of large numbers of starch grains, and how they differ from ordinary starch grains is somewhat difficult to see or to understand.

An examination of the ALMOND MEAL BISCUITS showed a similar state of things. Whether such preparations should be used in cases of glycosuria is a matter for the physician to decide on the merits of each individual case; from a chemical standpoint there is apparently no special merit appertaining to such diabetic foods as these, and they are decidedly misleading and injurious to those who require a rigid anti-diabetic dietary. It is stated that the object aimed at has been to so treat the starch that in the body of the diabetic it should behave differently from ordinary starch. Experience has shown us that the starch present in these foods becomes converted into diabetic sugar and is freely eliminated in the urine.

Two varieties of the CHELTINE SOLUBLE MILK FOOD are made, No. 1 and No. 2. The former is for infants from birth to three months, the latter for infants from 3–6 months and for invalids. Both preparations were subjected to careful chemical and micro-chemical analysis. They are both quite free from starch, and contain large amounts of proteid, milk fat, and milk sugar. They are partially soluble in water, forming an opalescent solution. From their composition they should prove an acceptable addition to the food of young children and also of adult invalids.

---

**Reynolds' Wheatmeal Brown Bread.**—J. REYNOLDS & Co., Gloucester.

A sample of this bread was shown at the Exhibition of the British Medical Association at Oxford, and another has been forwarded to us for examination. It is made entirely from wheatmeal, and is free from any mixture of salt or other chemicals. The wheat is of the best quality, and the loaf forwarded was a very good sample of wholemeal bread. It bears the alternative name of Reynolds' Gold Medal Digestive Brown Bread, and can be obtained from any baker in Great Britain.

---

**Meat Juice; Chicken Broth; Brand's A1 Soups.**—BRAND & Co., Vauxhall, S.W.

BRAND'S MEAT JUICE. (*Vide* vol. xi., 1893, p. 279.)—Analysis of this meat juice shows it to be one of the best of its kind. It shows well-marked absorption bands of oxy-hæmoglobin when examined spectroscopically, indicating that it has been prepared without heat. Coagulable proteids to the extent of approximately 26 grains in the fluid ounce are present, the proteid being dried at 100° C. before weighing. Extractives and salt in small quantity are also found in solution. It is worthy of note that such an extract as this acts not only as a stimulant, but also as a nutritious body.

---

**Trophonine,** a liquid food; **Zymocide,** a non-poisonous antiseptic; **Peptenzyme Elixir,** an agreeable digestive.—REED & CARNRICK, 80 Gloucester Road, South Kensington, London.

TROPHONINE is a solution of peptonised proteid with a pleasant flavour. On distillation it yields small quantities of alcohol, but there is no evidence of the presence of coagulable proteid. It is stated to contain animal and vegetable ferments, but its digestive power on coagulated albumen seems to be very slight indeed. It is palatable, non-irritating, requires no digestion, and leaves no residue.

The ZYMOCIDE is an elaborate and colourless fluid compound containing the following substances:—Extract golden seal, extract calendula, sulpho-carbonate of zinc, extract witch hazel, boracic acid, thymolate of soda, menthol, oil of wintergreen, oil of spearmint, and oil of eucalyptus.

It is believed by its originators that this fluid represents the latest advancement in chemical science and pharmaceutical skill in this important field of research, and that it is the perfection of all non-poisonous antiseptics. It forms a clear solution in water, is slightly alkaline in reaction, does not coagulate albumen, and undiluted has a germicidal power equal to a 10 per cent. solution of carbolic acid, or a 1 in 2,000 solution of mercuric perchloride.

PEPTENZYME is a combination of the active principles of many glands, including the salivary, peptic, pancreatic, and those of Lieberkühn and Brünner. It is said that the various digestive ferments contained in Peptenzyme do not interfere with, destroy, or injure each other. The Elixir is given in doses of from one to four drachms.

---

**Keystone Beef Wine; Keystone Burgundy.**—STEPHEN SMITH AND Co., Bow, London.

The KEYSTONE BEEF AND MALT WINE is the only one in which the Liebig Company's extract, Lemco, is used. It contains a small percentage of extract of malt, and has as its basis a light port. It is a sweet wine, with no unpleasantness of flavour, intended for those who cannot take ordinary food, and who require a nourishing and palatable stimulant.

The BURGUNDY is a natural unmedicated wine, ferruginous and full bodied.

---

**Vino di China Serravallo ferruginoso,** Serravallo's Tonic Wine.—J. SERRAVALLO, Pharmacist, Trieste. London: 46 Holborn Viaduct, E.C.

This is an excellent example of a mildly ferruginous wine, which apparently contains no added iron compounds. The preparation also contains the cinchona alkaloids, but not in sufficient amount to render the taste disagreeable. The amount of absolute alcohol found on examination is 12 per cent. by volume.

A liqueur glass after meals is the dose recommended by the proprietor. We have found that patients like it and ask for more.

---

**Granuloids.**—Buxton & Co., Clifton.

Bismuth Compound.—Each drachm contains a suitable dose of pepsine aseptic and bismuth carbonate in combination with gr. $\frac{1}{20}$ of morphia hydrochloride.

Aspirin.—Each drachm contains ten grains of aspirin (soluble). This is a very convenient form for the administration of this very useful anti-rheumatic and analgesic drug. A ten-grain dose may be repeated every two hours until relief is obtained. A few doses will usually give relief, even although the neuralgic pains may be dependent on the presence of organic disease, such as malignant growths.

---

**Bismutose,** an albuminate of Bismuth.—Kalle & Co., Biebrich-on-Rhine.

This new bismuth preparation is a pale-yellow amorphous powder, devoid of taste or smell. It is quite insoluble in water, in which, however, it swells up and forms a sort of emulsion, which settles very slowly. It is given in doses of 15 grains or more, either suspended in water alone, or with gum arabic or sugar. Clinically it has given excellent results, and it is claimed for it that the objections which have been alleged to exist against the use of the older bismuth preparations are absent when this new one is employed.

---

**Protylin "Roche"; Eisen-Protylin "Roche"; Bromo-Protylin "Roche"; Iodo-Protylin "Roche."**—Hugo Lorenz, 7-8 Idol Lane, London.

These manufactures of Messrs. F. Hoffmann, La Roche & Co., of Basle, Switzerland, are proteid bodies containing a group of phosphorus apposed to natural albumin. Protylin is a white, almost odourless and tasteless powder which is insoluble in water. It contains 2.7 per cent. of phosphorus, which is equal to 6.16 per cent. of phosphoric acid anhydride $P_2O_5$, and 12.98 per cent. of nitrogen. It resists artificial peptic but not pancreatic digestion; it is soluble in alkalies. Dr. A. Kocher reports "that protylin seems to fulfil the theoretical assumptions that, at all events in cases where it is a question of a supply of phosphorus, protylin is in its effect far superior not only to the usual inorganic compounds containing phosphorus, but also to the recently-recommended lecithine, and that protylin is perfectly non-toxic."

Further clinical evidence will doubtless be forthcoming with regard to these interesting chemical compounds; they appear to be worthy of continued investigation, and possibly may prove to be of great clinical value.

---

**Sels de Vichy-Etat.**—Sels minéraux naturels pour Boisson, 24 Boulevard des Capucines, Paris.

**Giesshübler Mineral Water; Insalus Mineral Water; Contrexéville Water.** Source du Pavillon.—INGRAM AND ROYLE, London.

Samples of these have been forwarded; they are well known, the least so is the Spanish one called Insalus. They are all alkaline waters, and have their uses in counteracting acidity and the uric acid diathesis.

---

**Colchi. Sal.**, Colchicine-Methyl-Salicylate.—ANGLO-AMERICAN PHARMACEUTICAL COMPANY, Croydon.

Each of these elegant capsules contains 20 centigrammes, and is equal in effect to 5 grains of sodium salicylate.

In acute inflammatory cases of gout, Laborde's method is especially recommended. This consists in administering one capsule, to begin with, every quarter of an hour until four capsules are taken; then an interval of three hours should be allowed to elapse, and again one capsule taken every quarter of an hour until another four are taken. Repeat this every three hours until sixteen are taken during the day. These doses should not be exceeded except in special cases.

On the second day, one capsule every quarter of an hour until three have been taken, then allow an interval of three hours, and continue on same lines until twelve are taken during the day.

On the third day, if the pain and swelling have not entirely subsided, the dose may be reduced to eight or ten during the day, and continued with still further reduction until the fourth or fifth day. Usually this is not necessary, but small doses of four capsules will in any case be sufficient to prevent relapse. We have found these capsules to be very effective in the treatment of acute gout.

---

**Sphagnol Soap and Ointment.**—71a Great Russell Street, London, W.C.

These peat products are said to be of considerable value for detergent and antiseptic purposes. The Sphagnol Medical Soap has a tarry odour and is almost black; it contains 15 per cent. of sphagnol, a product made by distillation of peat.

The colour and odour must prevent these preparations from attaining any popularity for ordinary toilet purposes, but when a tarry antiseptic is required the Sphagnol Soap and Ointment should be useful.

We are told that a touch of the ointment works wonders in wounds, burns and insect bites, and that travellers in the dry and dusty East swear by Sphagnol, which will make its way without meretricious puffing.

---

**Tabloids.**—Burroughs, Wellcome & Co., London.

Donovan Solution, min. 5.—The conditions in which arsenious iodide is usually prescribed are generally of such a nature as to demand more or less protracted treatment. "Tabloid" Donovan Solution has been introduced to provide a reliable and convenient means of administering this agent, and presents such a degree of compactness that a bottle containing 100 can be comfortably carried in the waistcoat pocket, and hence doses may be taken regularly when the patient is following ordinary pursuits. Each represents min. 5 of Liq. Arsenii et Hydrargyri Iodidi P.B., containing arsenious iodide and mercuric iodide, of each gr. $\frac{1}{22}$. The dose is one to four, dissolved in a wineglassful of water.

Ferric Chloride, min. 10.—This will be found an extremely convenient means of administering ferric chloride, especially to patients requiring a regular course of treatment. By employing the "Tabloid" preparation the drawbacks associated with carrying fluid mixtures containing ferric chloride are overcome. Each represents the amount of ferric chloride contained in min. 10 of Tincture of Ferric Chloride, B.P., and one dissolved in a large wineglassful of water may be taken as may be necessary.

Quinine and Strychnine (Quinine Bisulphate, gr. 1; Strychnine Sulphate, gr. $\frac{1}{60}$).—An excellent combination in the many and varied febrile conditions in which quinine is so largely prescribed. In such the antipyretic, antiseptic, and tonic effects of quinine are most marked, whilst the cardiac weakness and depression present in asthenic febrile states—a depression which the use of quinine alone might increase—are counteracted by the stimulating powers of strychnine.

For the loss of appetite and impairment of digestion consequent on fever, or present in conditions of anæmia, neurasthenia, &c., this "Tabloid" product, with its small dose of each constituent, forms an admirable gastric tonic. The dose is one to three, as may be necessary.

---

**Soloids.**—Burroughs, Wellcome & Co., London.

Ehrlich Triple Stain.—The mixture of one soloid dissolved n 25 c.c. of distilled water, with one soloid acid fuchsine in

2 c.c. of distilled water, forms a solution of the Biondi-Ehrlich-Heidenhain Triple Stain, which is ready for use and keeps well.

In specimens properly prepared the erythrocytes should be coloured orange, the granules of the neutrophile polymorphonuclear cells and of the neutrophile myelocytes should have a violet, and the acidophile granules of the polymorphonuclear cells a brick-red colour.

Ferric Chloride, gr. 10.—The advantages of this preparation are obvious. A solution of ferric chloride carried in the ordinary way is often the cause of annoyance from the liability of the containing bottle to leak or break. Vexatious stains or even considerable damage may thus occur. "Soloid" Ferric Chloride provides a means by which such contingencies may be entirely avoided. Each represents the amount of ferric chloride contained in 40 minims of Solution of Ferric Chloride, B.P., and one, dissolved in a sufficient quantity of water to produce 40 minims, forms a solution equivalent to the official strength. By varying the quantity of water this strength may be increased or diminished according to circumstances.

## Figures and Tables

**Figure f1:**